# Ophthalmology Versus Optometry

**Published:** 1904-03

**Authors:** 


### Ophthalmology versus Optometry.

THE annual struggle with its exchange of compliments is renewed. The opticians are trying to obtain a professional status from the legislature without having any recognised diploma or any definite course of study. In order to allay suspicion the medical fraternity is to be exempt from provisions of the bill creating this professional standing, thus acknowledging the right of medical men to the field the opticians are trying to invade.

A few years ago a horoscope, published in a jewelers journal, predicted that the care of the eyes and the adjustment of glasses would ultimately be entirely in the hands of the medical men. In the meantime that city is insignificant which has not at least one optical college. The founding of an institution of this sort is simplicity itself. The promoters decide who shall teach, the amount of fees, where to advertise, and the thing is done. The opticians have annual meetings where assemble those who style themselves professors of optics (from these colleges), the alumni (sic) of the institutions, together with others holding beautiful diplomas certifying they have paid from $7.50 to $10.00 for a correspondence course in optics, whereby they have learned such words as astigmatism, homonymous diplopia and one or two others equally impressive.

Ninety per cent. of the oculist's work is refraction, while very little space in the average ophthalmological journal is devoted to it. Where then can a medical man learn refraction? It is conceded that German, English and French medical institutions do not bother with the same nicety of adjustment of glasses that is found in this country, while here the places are lamentably few where one can learn refraction with any degree of thoroughness. How then is one to take up the work? During his college course he is shown a few sore eyes and is allowed to witness some eye operations performed by the attending ophthalmologist. He usually cuts these lectures to attend quizzes on pneumonia or therapeutics, for the importance of the eye in general medicine is rarely sufficiently emphasised. Later, if he becomes interested in eye work, he studies some books on refraction and tries to prove his work by the test case; but it takes him years in this way to learn what he could take up with the rest of his medical studies. The only other alternative is to attend some post-graduate course where he would get plenty of theory but, generally speaking, very little opportunity for practical experience in the work.

Articles have occasionally appeared objecting to opticians practising medicine by fitting glasses. If this is a department belonging to medicine, why isn't it included in the curriculum of medical colleges? If this is the case the colleges then should offer better facilities for the study of this branch of medical science and every graduate would be able to eliminate eye strain as a cause of reflex disturbances, to say nothing of the tremendous advantage a knowledge of the fundus of the eye would be as an aid in diagnosticating many general pathological conditions. More attention to the eye in the medical course would speedily settle for all time the controversy now going on, and keep the adjustment of glasses where it belongs,—under the care of licensed physicians.

